# Long-term side effects of testicular cancer and treatment (observational study of mortality and morbidity in testicular cancer survivors)

**DOI:** 10.1007/s00520-025-09447-0

**Published:** 2025-04-24

**Authors:** MRaheel Khan, Patrice Kearney Sheehan, Ashley Bazin, Abdur Rehman Farooq, Christine Leonard, Umair Aleem, Lynda Corrigan, Ray McDermott

**Affiliations:** 1 Department of Medical Oncology, Tallaght University Hospital, Tallaght, Dublin, Republic of Ireland; 2 School of Medicine, University College Dublin, Belfield, Dublin, D04 V1 W8 Ireland; 3 Department of Medical Oncology, St. Vincent’s University Hospital, Elm Park, Dublin, D04 YN26 Ireland; 4 Department of Medical Oncology, The Christie NHS Foundation Trust, 550 Wilmslow Road, Withington, Manchester, M20 4BX UK; 5 Department of Oncology, Princess Margaret Hospital, Toronto, Canada

**Keywords:** Testicular cancer, Survivors, Late side effects, Germ cell tumours, Cancer survivors

## Abstract

**Purpose:**

Testicular cancer (TC) is a rare cancer, but due to early age at diagnosis and excellent cure rates, there is a large cohort of survivors. Recent studies have highlighted the late side effects of treatments of TC, especially cisplatin-based chemotherapy. These complications make the survivorship care challenging with detrimental effects on health and prognosis of TC survivors (TCS). In this study, we provide a snapshot of common late side effects in TCS and a possible care pathway with a nurse-led specialised clinic.

**Methods:**

We invited TCS to participate in the study at one of the cancer centres in Ireland for a comprehensive screening using questionnaires, examination and blood tests. Further investigations were performed as indicated. Mortality was assessed through retrospective chart reviews.

**Results:**

We recruited 78 TCS to participate in the study with a median of 129 months since diagnosis (range 60 to 304 months) and 3 who died in survivorship. Second malignant neoplasms accounted for all three mortalities. Most common conditions after 5 years of diagnosis were hypertension (40%), dyslipidaemia (55.6%), hypogonadism (~ 45%), and high BMI (52%). The majority of conditions were diagnosed during screening, including two cases of coronary artery disease and one case of transient ischaemic attacks. TCS who received chemotherapy and were aged more than 30 years at the time of diagnosis had a higher prevalence of the late side effects.

**Conclusions:**

TC survivorship phase is marred by a range of late side effects. This remains a challenge for patients and healthcare workers as ambiguity surrounds the care pathways in the survivorship setting. We hope this nurse-led, specialised, screening clinic might improve the care and service provision for TC survivors.

**Supplementary Information:**

The online version contains supplementary material available at 10.1007/s00520-025-09447-0.

## Introduction

Testicular cancer (TC) is a rare solid organ tumour (1%) which usually occurs in the early years of life, most commonly between the ages of 18 to 39 years [1]. Recent studies have reported an increase in incidence with improvement in cure rates of TC in most developed countries including the US and North-Western Europe [2]. Therefore, we have a large cohort of TC survivors (TCS) increasing in size with each passing year. Although the cure rates are excellent, the treatment involves multiple modalities including high doses of cisplatin-based chemotherapy, surgical orchiectomy and radiotherapy. Late side effects of these treatment strategies have been under investigation for a long time [3]. Subsequently, oncologists have radically modified the treatment plans in most patients. Active surveillance after surgery without any systemic therapy or radiotherapy is becoming more popular in early-stage low-risk disease. Cisplatin-based chemotherapy still remains the mainstay of treatment in advanced and recurrent disease as novel treatments have not proven their efficacy yet [4]. The excellent cure rates in TC come at a price of late side effects in survivors [3, 5]. These side effects include a wide range of complications causing significant morbidity and early mortality in TCS. On one hand, these conditions cause early and premature mortality [6, 7], while on the other hand, they cause significant distress and detrimental effects on physical, social, sexual, economic, and psychological health [8–10].

Most commonly reported late side effects include second malignant neoplasms, late recurrence of TC, metachronous TC, cardiovascular disease, pulmonary disease, hypogonadism, metabolic syndrome, renal insufficiency, neurotoxicity, ototoxicity and Raynaud’s phenomenon [5–8]. Psychosocial sequelae include anxiety, fear of cancer recurrence, risk of suicide, infertility, unemployment and financial toxicity [11–13]. In this study, we focus on the comorbidities in TCS after 5 years of diagnosis, causing mortality and morbidity in this group.

This study is the first attempt to understand the TCS cohort in Ireland. Ireland has one of the highest incidences of TC worldwide [14] with treatment strategies similar to other European countries and the US. We conducted our research in one of the main tertiary care hospitals treating TC. The purpose of this study was to assess the point prevalence of late sequelae in TCS and also to provide a model of care. We believe this is the first study of its kind which provides comprehensive data based on clinical assessment of patients and also helps to establish care pathways for TCS. This article is the expanded version of the abstract published in the American Society of Clinical Oncology Journal as part of the annual meeting in June 2024 abstracts [15].

## Methods

### Study population

We identified all patients who had received treatment at Tallaght University Hospital in Dublin, which is one of the tertiary care cancer hospitals in Ireland for testicular cancer. Our records encompassed patients who had received treatment since 2006, which is the time when our department started maintaining a database for testicular cancer patients. Patients who had low-risk stage I disease followed up with the Urology Department instead of Medical Oncology, and hence were not included in our database. A research clinic was set up to perform an assessment on these patients. All patients who had crossed the 5-year (60-months) mark since diagnosis were invited to become part of the study. Only participants of age 18 and above at diagnosis were included in the study. In addition, the patients who had died after 5 years of diagnosis were also included in the study. Data was collected from February 2023 to February 2024. No exclusions were made based on treatment modalities; we included all patients, including those who underwent orchiectomy only.

### Ethics and good clinical practice

The study was granted approval in full by JREC (Joint Research and Ethics Committee) of Tallaght University Hospital, Dublin, Republic of Ireland. A written informed consent was obtained from the participants according to the ethics guidelines. Mortality data was collected through retrospective chart review. All staff involved in the study were trained in ethics, data protection, and Good Clinical Practice. Data was pseudo-anonymised at the time of collection. Complete anonymity was maintained during data processing and analysis.

### Assessment

A comprehensive assessment was performed in our clinic, including symptoms review, systemic inquiry, clinical examination and blood tests. A retrospective review of medical charts, radiology and pathology reports was done to establish the initial diagnosis of germ cell tumours, treatments received and previously known co-morbidities.

All second malignant neoplasms (SMNs), late recurrences of TCs and metachronous TCs reported in our study were diagnosed on the basis of pathology and radiology. Diagnosis of cardiovascular complications and hypertension was made after further investigations and assessment by the cardiology team. Low testosterone, high FSH (follicle stimulating hormone)/LH (luteinizing hormone), abnormal high glucose, lipids and impaired renal function were based on blood tests performed in the clinic. Hearing impairment was confirmed with audiograms in all cases.

HADS (hospital anxiety and depression scale) scores were also included in routine assessments to determine the levels of anxiety and depression in our cohort.

### Statistical analysis

Medical conditions known to the patients before diagnosis of cancer were excluded. All comorbidities which developed since cancer diagnosis were divided into known or newly diagnosed during the assessment in our clinic. We analysed these comorbidities as a percentage of total patients to provide point prevalence in our cohort. Comorbidities were analysed and reported in the groups stratified on the basis of treatment modality for TC, pathology of TC (seminoma vs non-seminomatous germ cell tumours) and age at diagnosis (below 30 years Vs above 30 years), according to relevance.

## Results

**Fig. 1:**
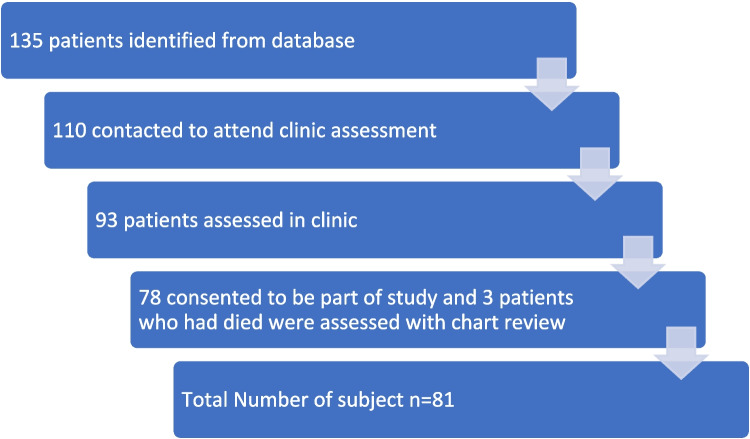
Number of patients inducted in the study

We identified 135 patients fulfilling the inclusion and exclusion criteria of our study. We attempted to contact all of them, but only 110 patients responded. For some patients, the contact information was outdated, and others did not respond. A total of 93 patients agreed to be assessed in our clinic after a telephone conversation regarding our clinic, while 17 of them refused to attend. Out of those, 81 patients consented to be part of the study (Fig. 1), while the remaining patients declined to allow us to use their data for research purposes.

The age at the time of assessment ranged from 21 to 72 years, with a median of 43 years, and the age at initial diagnosis ranged from 16 to 61 years, with a median of 33 years. The median duration from diagnosis to assessment was 129 months (10.7 years), with a range of 60 to 304 months. All of the participants had germ cell tumours, more than half of them (57%) classified as non-seminomatous germ cell tumours (NSGCT) and the rest (43%) as seminomas (Table 1).

**Table 1:** Demographics of patient cohort

Demographics of participants	
Age distribution Median age at diagnosis(range) Median time since diagnosis (range)	33 (16–61) years 129 (60–304) months
Pathology NSGCT *n*(%) Seminoma *n*(%)	46 (57) 35 (43)
Stage at diagnosis Stage I *n*(%) Stage II *n*(%) Stage III *n*(%)	44 (54) 19 (23) 18 (22)
Treatment Active Surveillance *n*(%) Chemotherapy *n*(%) Radiotherapy *n*(%) RPLND *n*(%) Orchiectomy *n*(%)	26 (32) 69 (85) 4 (5) 14 (17) 80 (99)
Chemotherapy regimen BEP1 BEP3 BEP4 EP4 VIP4 BEP3EP1 Carbo1 Other (HDCT, TICE)	*n* = 69 (%) 4 (6) 11 (16) 14 (20) 19 (28) 3 (4) 4 (6) 3 (4) 12 (17.4)

On initial presentation, 54% of patients were stage I, 23% stage II and 22% stage III cancer. Due to selection bias mentioned in study limitations, these findings are in contrast to the data maintained by National Cancer Registry of Ireland. According to the national database, 69% patients presented with stage I, 16% with stage II and only 10% with stage III [16].

Every patient underwent orchiectomy except for one, who presented with extra-testicular germ cell cancer. Active surveillance after orchiectomy was opted for in 26 (32%) patients, but unfortunately, 22 (85%) of them had a recurrence of disease after a median duration of 14 months. A significantly higher number (85%) of our study population had received chemotherapy due to unintentional selection bias explained in the limitations section below. Of those 69 patients who received chemotherapy, 11 received 3 cycles of BEP (bleomycin, etoposide and cisplatin), 14 had 4 cycles of BEP, 19 had 4 cycles of EP (etoposide and cisplatin), and 4 had 4 cycles of VIP (etoposide, ifosfamide and cisplatin). In the adjuvant setting, 1 cycle of carboplatin was given to 3 patients, while 1 cycle of BEP was given to 4 patients. Details of treatments are mentioned in Table 1. Radiotherapy was used in 4 (5%) patients, including one patient who received whole brain radiotherapy for brain mets. Retroperitoneal lymph node discussion (RPLND) for residual masses after chemotherapy was performed in 14 (17%) patients. No cases of primary RPLND in stage II disease were identified.

**Fig. 2:**
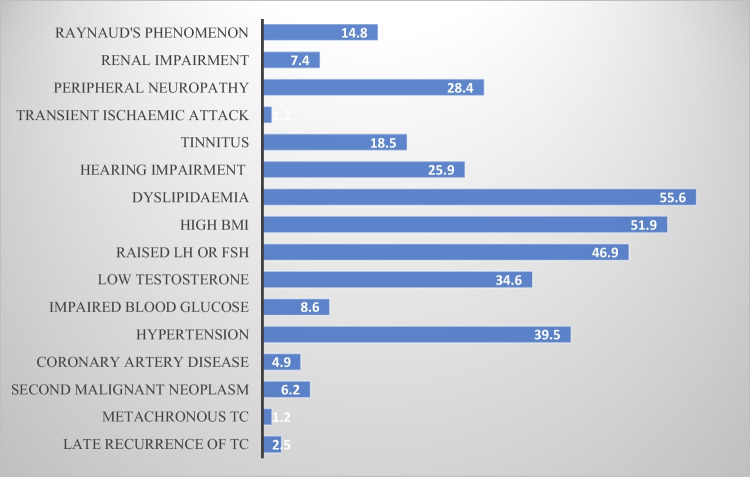
Prevalence (%) of late side effects in all TC survivors

We have summarised the prevalence of these sequelae in Fig. 2. The details and discussion on these complications are reported in separate headings.

We identified five (6%) cases of SMNs, occurring at a median of 109 months (9 years) from diagnosis of TC. Chemotherapy had been used in all of these patients; none of them received any radiotherapy. Patients who developed SMNs were all aged 30 years or more at the time of TC diagnosis. All 3 deaths in our TC survivors were caused by SMNs. Malignancies included lung cancer, colon cancer, prostate cancer, oesophageal cancer and leiomyosarcoma. Our data showed one case of metachronous TC (1.2%). The patient was initially diagnosed with seminoma, treated with orchiectomy and radiotherapy, subsequently developed NSGCT after 7 years and underwent orchiectomy and chemotherapy. On follow-up after 14 years since the second TC, no signs of recurrence were found. Two (2.4%) cases of late recurrences of TC (after more than 5 years of diagnosis) were noted with a mean duration of 72 months (6 years) since the initial diagnosis. One of them had NSGCT treated with surgery and chemotherapy, while the other one had seminoma initially diagnosed at stage IB and treated with surgery only. NSGCT recurred as a teratoma, which was surgically resected, and seminoma was treated with chemotherapy. Both patients were disease-free at the time of follow-up which was more than 5 years since recurrence.

In this study, four (5%) patients were found to have coronary artery disease. Half of them were already diagnosed, but the other half was only diagnosed during their assessment in the clinic. All of these patients (100%) had received chemotherapy and were aged more than 30 years at the time of TC diagnosis. No cases of cardiomyopathy were found in our study. Interestingly, 2 of these 4 patients were not aware of their underlying coronary artery disease. Systemic enquiry by our researchers led to further investigations by our cardiology team, which in turn diagnosed and managed their condition. This finding emphasises the need for screening clinics to reduce the mortality as a consequence of cardiovascular complications in TCS. Hypertension was found to be the second most common underlying condition in our study, with almost 40% of patients affected. The median duration to develop hypertension was 109 months (9 years), but only one-third of these patients were diagnosed in primary healthcare, and the rest were diagnosed in our clinic. Chemotherapy was used in 81% of the cases, and 75% were aged more than 30 years at the time of diagnosis.

**Fig. 3:**
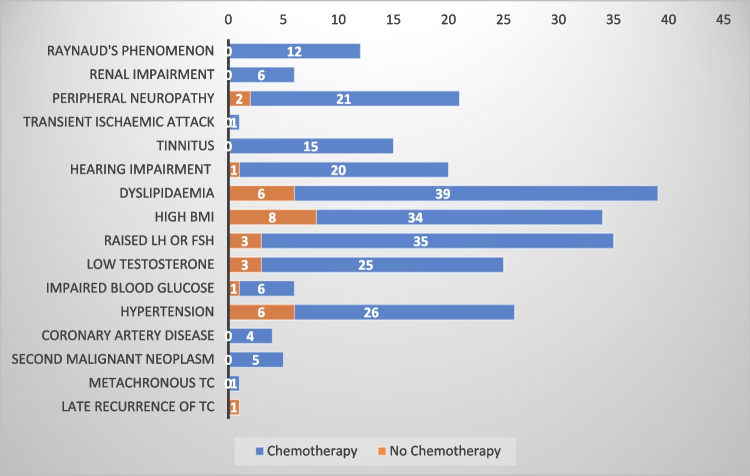
Prevalence of late side effects in the patients who received chemotherapy vs no chemotherapy

Our assessment revealed abnormally high glucose levels in seven (22%) patients, based on fasting glucose and HbA1c readings. Of those seven patients, six had received chemotherapy for treatment of TC and five were aged more than 30 years at diagnosis (Figs. 3).

Body mass index (BMI) was calculated based on weight and height measurements in clinic. We found high BMI in 42 (52%) patients with the majority (81%) belonging to the chemotherapy group. The internationally recognised range of normal BMI was utilised for assessment using height and weight measured in the clinic [17].

More than half (51%) of our patients had abnormal high levels of cholesterol or triglycerides on blood tests. Only a small minority (13%) were aware of this underlying condition, as most of them were diagnosed on screening. Dyslipidaemia was clearly predominant in the patients who received chemotherapy, accounting for more than 85% of patients. It was also more common in TCS diagnosed at > 30 years of age.

We assessed serum testosterone and FSH/LH levels in all of our patients. An abnormal low-serum testosterone was subsequently confirmed with early morning fasting levels. We found low levels of testosterone in 28 (34.5%) and an abnormal high FSH or LH in 38 (47%) patients. Patients who had received chemotherapy accounted for almost all cases of gonadal dysfunction, representing almost 90% of this cohort. Even with a high prevalence of symptoms, only 17% of patients were aware of their underlying condition.

In this cohort, the prevalence of hearing impairment was 29%, almost exclusively in the patients who received chemotherapy (95% of cases). Like in all other comorbidities, the majority of cases (76%) were diagnosed during screening in our clinic. Similarly, tinnitus was reported by 15 (18.5%) patients, and all of them had received chemotherapy.

Only one of our patients was diagnosed with transient ischaemic attacks after detailed investigations of symptoms. No strokes were detected or reported in our study. On the contrary, 23 (28%) patients reported residual peripheral neuropathy after a median follow-up of 8.5 years. This assessment is solely based on patient-reported questionnaires since we could not perform any further investigations like nerve conduction studies to confirm the diagnosis.

In this study, we did not find any cases of pulmonary disease diagnosed after TC diagnosis or treatment. Even though 42 (52%) of our patients had received one or more cycles of bleomycin.

We found six (7.4%) patients with persistent renal impairment after a median follow-up of more than 10 years. Of note, only one out of these six patients had an established diagnosis of renal compromise. The rest of the five patients were diagnosed during screening in our clinic and later confirmed by the nephrology team. Similar to ototoxicity, nephrotoxicity was only found in patients who received chemotherapy.

At the median follow-up of 12 years, Raynaud’s phenomenon was reported by 12(15%) patients, all of whom received chemotherapy for treatment of TC. Both patients diagnosed with coronary artery disease in our clinic had Raynaud’s phenomenon as well, which underscores the close relationship between the two complications.

Out of 78 patients we assessed in our clinic, 76 filled out HADS (Hospital Anxiety and Depression Score) pro forma. An internationally accepted scale was used to interpret HADS results [18].

We found 17% of patients with mild anxiety and an additional 14.5% with moderate and severe anxiety. Only 8% of patients reported mild depression, and one respondent reported moderate levels.

## Efficacy of screening clinic in TCS

**Fig. 4:**
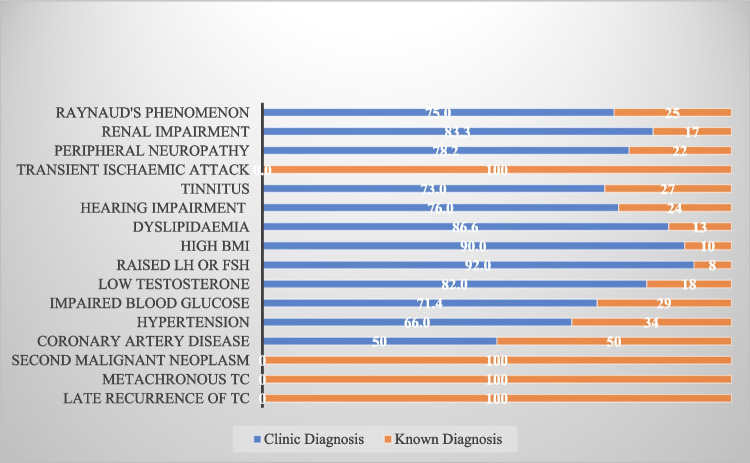
Conditions diagnosed during screening in clinic compared to known comorbidities

We have attached our screening proformas in the supplementary material. Our nurse-led research clinic can serve as a model for survivorship care in TCS. As shown in Fig. 4, the majority of the late complications were diagnosed in the clinic during assessment. Further investigations under a specialist team such as cardiology and endocrinology were performed for abnormal findings during screening.

Kvammen et al. have featured the causes of inferior survival in TCS with their registry-based study [7]. SMNs followed by cardiovascular complications were identified as the most common cause of early death in TCS. In our study, we have established that most of the cardiac risk factors, including hypertension, obesity, diabetes, and hypogonadism, can be detected and corrected with regular assessment of TCS. In this way, early deaths due to cardiac complications can be prevented. Lubberts et al. also noticed that when cardiac risk factors were treated, no excess risk of cardiac complications was found [19]. In the same way, regular symptom checks and clinical examinations might be able to identify early signs of malignancy and reduce the mortality related to SMNs. We do believe that a more comprehensive strategy is needed for early detection of solid tumours. A good example is early screening for breast and prostate cancer in patients with BRCA mutations.

## Discussion

Regarding second malignancies, our findings are similar to the other studies investigating the same in TC survivors. Kvammen et al., in a wide, registry-based analysis, also reported SMNs as the commonest cause of death in TCS after 5 years [7]. The 25-year cumulative incidence of solid SMNs in TCS was reported at 10% by Groot et al., which is much higher than our observation [20], but our cohort is relatively much younger, with a median duration of around 10 years since diagnosis. Similar types of malignancies have been reported by other investigators [20, 21], and a higher prevalence with the use of chemotherapy and a higher age at diagnosis is also widely reported in the literature [7].

Mrinakova et al. performed a retrospective study to investigate the incidence of second TC in 2124 patients between 1970 and 2020 [22]. Overall incidence was reported at 4.1% in all cases after a median duration of 6.7 years in seminoma and 9.2 years in NSGCT patients. Similar to SMNs, our observed number is lower (1.2%) compared to their findings. Firstly, as mentioned earlier, our cohort is still young, and this incidence could rise in coming years. Secondly, we observed a disproportionately high percentage of patients who had received chemotherapy due to the reasons mentioned in limitations below. Chemotherapy has been reported to reduce the incidence of second TC [22, 23].

Our patient with metachronous TC had initial cancer at the age of 31, contrary to Hellesnes et al.’s finding of higher incidence with age less than 30 [23].

Ondrusova et al. also reported a 2.6% relapse rate in their retrospective analysis [24]. Late relapses have been reported with both seminoma and NSGCT [25] with an interval of 6–7 years from initial diagnosis [24, 26]. Late recurrence of teratoma in the second patient after chemotherapy has also been widely reported in the literature [24, 26, 27].

Lubberts et al. assessed the incidence of cardiovascular disease in a multicentre cohort including 4748 TCS. They reported a 5.7% incidence at a median of 16 years of follow-up [19], very similar to our findings. Cisplatin-based chemotherapy and an older age have been repeatedly reported in studies as major risk factors [19, 28, 29]. As mentioned above, Lubberts et al. conducted a comprehensive study to evaluate cardiovascular risk factors in the TC population of the Netherlands [19]. They reported hypertension in 50% of 4787 TC survivors compared to 39.5% in our study. This difference can be explained by the contrast in median follow-up of both studies. The median follow-up in this Dutch study was 22 years compared to 10 years in our study. Cisplatin-based chemotherapy has been reported in other studies to increase the risk of developing hypertension [5, 30). A staggering 40% of TCS with abnormally high glucose levels were found in their study compared to 22% in our cohort [19]. Again, this discrepancy between the two studies could be due to a longer duration of follow-up (10 vs 22 years). Both studies followed similar methods of assessment and cut-offs for normal levels. Pre-diabetic levels of impaired glucose are more prevalent in this cohort compared to diabetes [8]. Due to study limitations, we are not able to report the proportion of diabetics in this cohort.

Many studies have reported a higher incidence of obesity and pre-obese high BMI after chemotherapy in TCS [5, 8, 19]. Kerns et al. assessed 1214 TCS who had received chemotherapy, in a multi-institute study. The study revealed 71.5% of TCS with high BMI with a median of 4.2 years since diagnosis [8]. Our study showed similar results with a high prevalence mainly in the chemotherapy group. Although a similar percentage (77%) was reported in all TCS by Agrawal et al. based on a questionnaire-based study conducted in the US, the chemotherapy cohort still had a much higher incidence [5]. While evaluating CVD risk factors in TCS, Lubberts et al. showed 85% of patients with dyslipidaemia [19]. Similar to differences in other results, a higher incidence in their study could be due to an older cohort of TCS (10 years vs 22 years of follow-up duration). As reported in our results, only 15% of TCS in this Dutch study were aware of their condition. In both cohorts, around 85% of patients were not conscious of their underlying disease.

Our study findings are comparable to the study on hypogonadism conducted in the UK by Huddart, et al. [31]. They evaluated hormone levels in 640 patients in the survivorship phase of TC. Low levels of testosterone were found in 34% of the patients in the chemotherapy and radiotherapy group compared to 11% in the surveillance group. High FSH in the surveillance and chemotherapy/radiotherapy groups was 42% and 70%, respectively. A similar trend was seen with raised LH (6% in surveillance group vs 11% in chemotherapy/radiotherapy group). We reported similar results in our cohort. Huddart also highlighted issues of infertility and sexual dysfunction along with these hormonal abnormalities; we have reported these previously in a questionnaire-based survey [32].

Numerous studies have investigated the ototoxicity related to cisplatin exposure during treatment of TC. The most commonly reported incidence is around 30–40% in TCS who received chemotherapy, similar to our results [5, 8, 33]. A rise in incidence with higher age at diagnosis was also reported in a US study [5].

We found 28% of patients with residual peripheral neuropathy, similar to 26.5% reported by Agrawal et al. for the US population of TCS [5]. The prevalence of Raynaud’s phenomenon (15%) was also close to their study which reported Raynaud’s in 18% of all TCS and 33% in the TCS with prior exposure to chemotherapy [5].

For pulmonary toxicity, the most notable study on this subject was performed by Haugnes et al., in Norway. Around 16–17% of long-term survivors were found to have compromised pulmonary function after chemotherapy treatment [34]. The results from our study (0%) are possibly a consequence of more cautious use of bleomycin in TC patients.

In the majority of studies evaluating renal impairment in TCS, the incidence is reported around 3% [5, 8]. A much higher incidence (23%) of reduced glomerular filtration rate has been mentioned after 1 year of cisplatin-based treatment [35]. Our study found 7.4% of patients with renal impairment.

The prevalence of anxiety in our study goes in line with the 20% prevalence of anxiety in TCS after 5 years reported by AB Smith et al. [36]. The systematic review by Smith et al. reported no increased risk of depression in TCS compared to the general population.

## Strengths and limitations

To our knowledge, this is the first research to provide a comprehensive care plan and a snapshot of every late side effect in all TCS. Previous studies providing prevalence and incidence in all TCS were mainly based on registry analysis or questionnaire-based surveys [5, 7]. Other studies evaluating patients with clinical assessment either focused on a group of patients [8] or a group of diseases [19].

In this study, we also propose a working model to improve the morbidity and mortality in TCS through nurse-led, purpose-built clinics with referral pathways in place. During our study, we noticed a great amount of interest from the survivors and the patient communities.

Although, at the time of conception, we intended to include all patients, including those who underwent surveillance only after surgery, but unfortunately, we noticed an unintentional selection bias in our study.

We only had access to the database maintained by the Medical Oncology Department of our hospital. Patients who receive orchiectomy alone, for low-risk stage I disease, are usually followed up by our surgical colleagues in the Urology Department, while high-risk stage I disease is referred to Medical Oncology for surveillance. This translated into a higher rate of recurrence in stage I patients and a higher proportion of patients who had received chemotherapy. Also, patients who had received chemotherapy or were experiencing any late side effects were more likely to attend our clinic and participate in the study. This selection bias dramatically affected our study outcomes, exaggerating the prevalence of late side effects and rates of recurrence.

A more accurate estimate could be possible with certain conditions like Raynaud’s phenomenon and peripheral neuropathy by using specialised investigations. Due to limitations in resources, we could not perform these investigations. Also due to similar constraints, we did not include morbidities known to patients before TC diagnosis, which could have been exacerbated by the treatment.

## Conclusion

The cure rates in TC have improved with multimodal treatment strategies, but the care for survivors remains a challenge. TCS are facing a spectrum of physical, psychological and social challenges after the cure of cancer. Serious deficiencies can be seen in the survivorship care as most cancer centres do not offer any follow-up at the end of surveillance. Our study provides the magnitude of difficulties and a practical solution. We are hoping that further studies will build up on this knowledge and improve the survivorship care as well as raise awareness among healthcare providers and survivors. Five decades ago, testicular cancer patients emerged as pioneers in solid organ cure; now, they can become the pioneers in survivorship care.

## Supplementary Information

Below is the link to the electronic supplementary material.

Supplementary file1 (DOCX 356 KB)

## Data Availability

No datasets were generated or analysed during the current study.
