# Supplementary material for: Long-term side effects of testicular cancer and treatment (observational study of mortality and morbidity in testicular cancer survivors)
Source: Support Care Cancer. 2025 Apr 24;33(5):413. doi: 10.1007/s00520-025-09447-0 (PMC12021710; doi:10.1007/s00520-025-09447-0)
Supplement: Supplementary file 1 — Supplementary file1 (DOCX 356 KB) [file 520_2025_9447_MOESM1_ESM.docx]

Name: _____________________________ DOB: ___________ Age: _______

Seminoma  Non-Seminoma : _____________________________________

Date of Diagnosis: __________ Age at diagnosis: ____________

Stage: T__ N__ M__ S__ Stage Group: _____

Orchiectomy: _____________ Side : ______

Chemotherapy: __________ EOT : ___________

Chemotherapy 2: ________________ N/A  EOT: ___________

RPLND: ______________ N/A  Radiotherapy: __________ N/A

Allergies: ____________

Weight: _______ kg Height: ______ cm BMI: _____ Kg/m^2^

**Co Morbidities: (With Dates)**

1._____________________ 5._______________________

2._____________________ 6._______________________

3._____________________ 7._______________________

4._____________________ 8._______________________

**Current Medications:**

1._____________________ 5._______________________

2._____________________ 6._______________________

3._____________________ 7._______________________

4._____________________ 8._______________________

**Smoking Status:**

Smoker  Ex-Smoker  Non-Smoker

**Alcohol Intake:**

Regular  Occasional Units/week: _________

**B Symptoms:**

Night sweats Yes  No Duration: _______

Unintentional Weight loss Yes No Duration: _______ Amount: ___Kg

Loss of energy Yes  No Duration: _______

**Cardiovascular Review:**

|  |  | Yes | No |
| --- | --- | --- | --- |
|  | Did you notice any recent change in exercise tolerance? |  |  |
|  | Do you feel any chest discomfort on exertion? |  |  |
|  | Do you feel any Shortness of Breath on Exertion? |  |  |
|  | Do you feel any shortness of breath on lying down? |  |  |
|  | Do you wake up at night feeling short of breath? |  |  |
|  | Do you get any swelling in your legs? |  |  |

**Respiratory Review**:

|  |  | Yes | No |
| --- | --- | --- | --- |
|  | Do you feel any difficulty in breathing? |  |  |
|  | Do you have any new persistent cough? |  |  |
|  | Do you hear any whistling sounds in your chest? |  |  |
|  | Do you find difficult to catch your breath? |  |  |
|  | Do you have any coughing at night? |  |  |

**Raynaud’s Phenomenon:**

|  |  | Yes | No |
| --- | --- | --- | --- |
|  | Are your fingers sensitive to the cold? |  |  |
|  | Do your fingers change colour in response to cold (white, red, blue)? |  |  |
|  | Do you get numbness or pain in the affected area when they change colour? |  |  |
|  | Do you feel stinging or throbbing when affected area warms up? |  |  |

Hearing Review:

|  |  | Yes | No |
| --- | --- | --- | --- |
|  | Do you experience any difficulty in hearing? |  |  |
|  | Do you hear any buzzing or ringing when its quiet? |  |  |

Testicular:

|  |  | Yes | No |
| --- | --- | --- | --- |
|  | Have you noticed any pain or tenderness in your testicles? |  |  |
|  | Have you noticed any tenderness in your testicles? |  |  |
|  | Have you noticed any abnormal swelling in your testicles? |  |  |

**Examination:**

**General Exam**:

BP: _____ Pulse: ____ Respiratory Rate: ____ Temp: _____ O_2_ Sats: ____

|  | Yes | No | Details |
| --- | --- | --- | --- |
| Pedal Oedema |  |  |  |
| Sacral Oedema |  |  |  |
| Raised JVP |  |  |  |
| Muscle wasting |  |  |  |

Lymph node exam:

| Region | Normal | Abnormal | Details |
| --- | --- | --- | --- |
| Peri-auricular |  |  |  |
| Sub-mandibular |  |  |  |
| Cervical |  |  |  |
| Supraclavicular |  |  |  |
| Axillary |  |  |  |
| Inguinal |  |  |  |

Cardiovascular:

|  | Yes | No | Details |
| --- | --- | --- | --- |
| Added heart sound |  |  |  |
| Irregular Rhythm |  |  |  |
| Other |  |  |  |

Respiratory:

|  | Yes | No | Details |
| --- | --- | --- | --- |
| Wheeze |  |  |  |
| Crepitations |  |  |  |
| Basal crepitation |  |  |  |
| Other |  |  |  |

Abdomen:

|  | Yes | No | Details |
| --- | --- | --- | --- |
| Palpable mass |  |  |  |
| Hepatomegaly |  |  |  |
| Splenomegaly |  |  |  |

Testicular exam:

|  | Yes | No | Details |
| --- | --- | --- | --- |
| Tenderness |  |  |  |
| Swelling |  |  |  |
| Prosthesis |  |  |  |

Blood Results:

|  | Value | Range | Date | Comments |
| --- | --- | --- | --- | --- |
| WCC |  |  |  |  |
| Hb |  |  |  |  |
| Platelets |  |  |  |  |
| Creatinine |  |  |  |  |
| Creatinine Clearance |  |  |  |  |
| Glucose |  |  |  |  |
| HbA1c |  |  |  |  |
| Total Cholesterol |  |  |  |  |
| Triglycerides |  |  |  |  |
| Serum Testosterone |  |  |  |  |
| LH |  |  |  |  |
| FSH |  |  |  |  |
| AFP |  |  |  |  |
| HCG |  |  |  |  |
| LDH |  |  |  |  |

**Impression:**

**Plan:**

HADS:


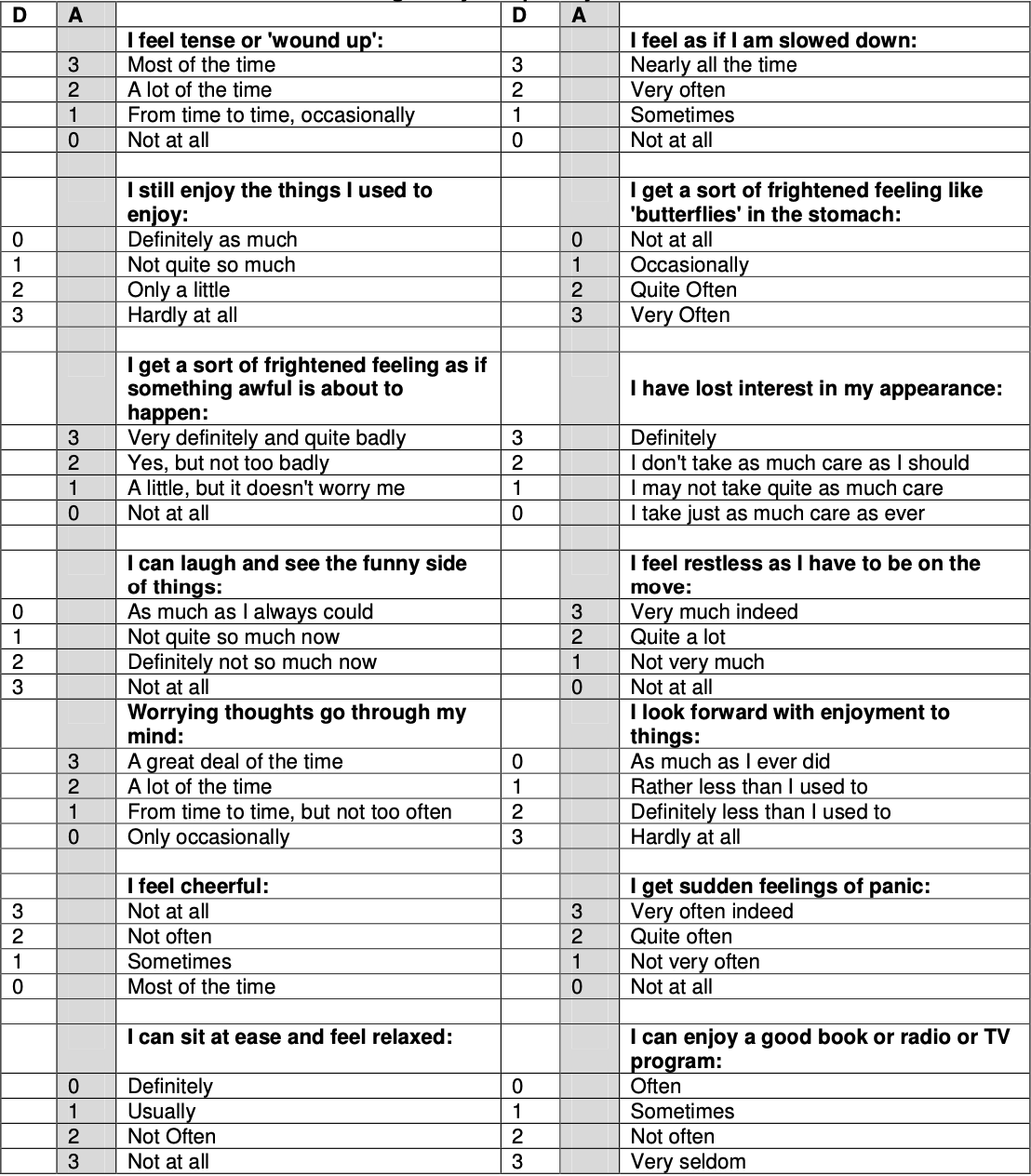


Anxiety Score: _______ Depression Score: ___________
